# Clinical Utility of Psychoeducational Interventions for Youth with Type 1 Diabetes: A Scoping Review

**DOI:** 10.5334/cie.28

**Published:** 2021-07-15

**Authors:** Lana Bergmame, Steven Shaw

**Affiliations:** 1 McGill University, CA

**Keywords:** diabetes, adolescents, psychoeducational interventions, clinical utility

## Abstract

Adolescence is a challenging time for the medical management of type 1 diabetes. Thus, a range of psychoeducational interventions have been developed to improve diabetes management among youth. Systematic reviews of this literature have emphasized the effectiveness of interventions for improving patient outcomes. However, knowledge beyond what works is required for interventions to be adopted into routine clinical practice. The objective of this scoping review was to map the clinical utility of the literature based on a variety of indicators, including the problem base, context placement, information gain, transparency, pragmatism, and patient-centeredness of the research. This lens for reviewing research is consistent with the biopsychosocial model and an increasing focus on reducing disability, including activity limitation and participation restriction. PsycINFO, MEDLINE, and CINHAL databases were searched for evaluative psychoeducational intervention studies published between January 2005 and October 2020. Two cited reference searches and one reference list search were also performed. Fifty studies describing 46 different interventions were identified. The clinical utility of the interventions was highly variable. A detailed overview of the clinical utility of the literature is provided with an emphasis on current gaps and shortcomings to be addressed in future research. This work helps advance the translation of clinical knowledge into practice in schools, homes, and communities; and, ultimately, improve the health and well-being of adolescents with T1D.

## Type 1 Diabetes for Adolescents

Type 1 diabetes (T1D) is one of the most common chronic medical conditions affecting youth (Naranjo & Hood, 2013). Approximately 107,300 North American and Caribbean children are currently affected by the disease, with about 16,500 new cases diagnosed annually (International Diabetes Federation, 2015). T1D is a complex and chronic disorder of the endocrine system, in which the body attacks its own insulin-producing Beta cells, necessitating the need for insulin injection (Canadian Diabetes Association, 2013). Individuals with T1D can experience serious and life-threatening health complications, including episodes of hypoglycemia, or dangerously *low* blood glucose levels, and diabetic ketoacidosis, resulting from dangerously *high* blood glucose levels (Canadian Diabetes Association, 2013). Having blood glucose levels that are too low or too high affects multiple systems of the body, and over time, leads to a range of serious health issues, including blindness, limb amputation and, in some cases, coma and death (Canadian Diabetes Association, 2013). These potential complications, and the vigilance required to prevent them, can also lead to psychosocial challenges, including increased personal and familial stress, feelings of depression and anxiety, and negative body image (Naranjo & Hood, 2013). Negative effects can be mitigated by effective medical and psychoeducational interventions (Hermanns et al., 2020; Murphy et al., 2012), however.

Medical management of T1D is complex and demanding. It involves daily insulin injections (or use of an insulin pump), frequent blood glucose monitoring, following a balanced diet, and regular physical activity (Canadian Diabetes Association, 2013). The goal for medical management of diabetes is optimal glucose control, which is typically defined as hemoglobin A1C (HbA_1c_) less than or equal to 7% or a mean blood glucose level of ≤8.6 mmol/L (Canadian Diabetes Association, 2013).

Achieving optimal blood glucose levels requires adherence to prescribed medical recommendations and mastery of a repertoire of self-care skills (Survonen et al., 2019). However, engaging youth in self-care activities is often challenging, and many do not achieve management targets (Katz et al., 2014). For example, over 40% of adolescents with diabetes do not carry out scheduled blood glucose monitoring and over 25% miss at least one insulin injection per week (Weissberg-Benchell et al., 1995). Approximately 70% fail to follow recommended dietary guidelines (Heinrich et al., 2010) and over 80% do not meet physical activity guidelines (Shalev & Geffken, 2015).

Poor medical adherence occurs at all ages, but adolescents present with a unique set of developmental challenges affecting diabetes care. Adolescence, in general, is a challenging developmental period characterized by rapid change and adjustment (Taddeo et al., 2008), and having T1D poses additional challenges. Poor medical adherence during this period has been attributed to several factors, including developmental conflicts between the need for independence and closeness with parents, the desire for peer acceptance and conformity, the presence of increasing incidence of mental health challenges, and typical adolescent rebellion related to the development of a self-directed identity (Hanna & Guthrie, 2003; Martinez et al., 2018).

During adolescence, shifts typically take place in the responsibility of care from the parent or caregiver to the child. During this transition, many adolescents experience an increasing need for independence, but the daily living skills required for this independence are still developing (Markowitz et al., 2015). Self-directed care requires a sophisticated skill set and strict adherence to treatment recommendations. Gaining independence in diabetes management, when adolescents may not be entirely ready to take on this responsibility, can lead to increased anxiety, which can be reduced through increased parental involvement (Markowitz et al., 2015). However, this increased parental involvement is in direct conflict with adolescents’ desire for independence and may lead to the refusal to adhere to prescribed treatment regimens (Shaw, 2001).

The need for peer acceptance, and desire for conformity with peers, can also have a substantial influence on adolescents’ diabetes self-management (Akhter et al., 2018). That is, having to follow a prescribed treatment regimen serves as continuous reminder to adolescents of the presence of their disease and may also alert peers to their illness. When faced with pressures to conform, adolescents often resist treatment recommendations (Shaw, 2001). School climate, culture, and supports in a school setting also play a role in the context of supporting diabetes self-management among adolescents.

Higher rates of mental health problems among adolescents with T1D have also been linked to poorer diabetes management (Naranjo & Hood, 2013). Specifically, youth with T1D are reported to have a three times higher prevalence of depression than peers without the disease (Grey et al., 2002), and depression has been associated with less frequent blood glucose monitoring and poorer glycemic control (McGrady et al., 2009). One factor contributing to this relationship may be perceived self-efficacy. That is, adolescents experiencing depression might feel that they have limited or no control over their diabetes, resulting in diminished motivation to initiate and carry out the necessary management tasks (McGrady et al., 2009). Adolescents with T1D are also at an increased risk for anxiety, with rates estimated between 13 and 17% (Herzer & Hood, 2010). Anxiety regarding future diabetes complications can negatively impact adolescents’ self-management and overall quality of life (Herzer & Hood, 2010). Mental health supports in the community and school have the indirect effect of increasing effective diabetes self-management (Carroll & Vittrup, 2020).

## The Shortcomings of Psychoeducational Interventions

Factors affecting adolescents’ diabetes self-care warrant consideration given that behaviours established during this period can have long-lasting effects on future health and well-being (Corathers et al., 2015). Thus, failure to master self-care behaviours places youth at a heightened risk for a range of medical complications and lower quality of life as they progress into adulthood (Corathers et al., 2015). As such, there is a need to improve adolescents’ self-management behaviours by targeting not only the acquisition of required skills but also the motivation to self-manage (Wiebe et al., 2018).

Psychoeducational interventions designed to improve adolescents’ adherence to medical regimens are integral to diabetes care (Abraham et al., 2018). Such interventions typically provide training in areas such as diabetes-related problem-solving, the development of communication and coping skills, as well as individual and family-focused therapy (Hampson et al., 2001). There is presently a large body of literature describing psychoeducational interventions for adolescents with T1D. However, comprehensive reviews of this literature have revealed mixed results in terms of intervention design and effectiveness (Bergmame & Shaw, 2018; Hampson et al., 2001; Hendrieckx et al., 2020; Murphy et al., 2006). For example, Bergmame and Shaw (2018) identified 42 evaluative studies of psychoeducational interventions for adolescents with T1D published over the past 12 years. Although their review highlighted recent progress in terms the quality and quantity of intervention studies, the measurable effects of psychoeducational interventions for a range of outcomes were modest at best (Caccavale & Monaghan, 2020).

## Clinical Utility and the Biomedical Model

A common overarching goal of this work is to inform clinical practices and positively influence patient outcomes. Achieving consistency with a biopsychosocial approach means going beyond simple effectiveness or efficacy to be comprehensive and integrative (Wade & Halligan, 2017). Care that allows adolescents to feel understood and empowered is critical to any success. Influencing self-care is an important goal, which involves sociocultural, psychological, systemic, and biological information. Effectiveness is the critical factor in the biomedical model, but clinical utility is the often-forgotten factor consistent with the biopsychosocial model of interventions. *Clinical utility* is not simply about what works. It is a multi-dimensional concept describing the usefulness and relevance of an intervention for clinicians (Lesko et al., 2010; Smart, 2006) and supporting complete conceptualization of the problems to be addressed with interventions that minimize unintended negative consequences and empower adolescents with T1D.

Interventions by themselves do not have inherent utility. Indeed, it is the implementation and sustainability of a given intervention that influences health outcomes. However, implementing clinical research often poses challenges to clinicians who must consider the applicability of the evidence to patients, the feasibility of the intervention in a school or community, and the potential overall influence on patients. Clinical research has been called into question for lacking utility in patient care (Ioannidis, 2016). To be clinically useful, it is not enough solely to consider the effectiveness of an intervention and the quality of the research design. Information regarding the appropriateness, feasibility, and acceptability of interventions is also imperative (Smart, 2006). To date, important questions remain regarding the usefulness and relevance of psychoeducational intervention research for youth with T1D in real-world settings consistent with the biopsychosocial approach.

There are consequences for effective psychosocial regimens for adolescents. Not only does poorly managed diabetes have functional and structural outcomes, such as loss of vision, peripheral nerve damage, or limb loss; it also has consequences for participation and activity. The limitations on physical activity, social life, community engagement, and full participation in culture have a dramatic effect on the lives of adolescents with T1D. The biopsychosocial model and clinical utility extend effectiveness to address how useful an intervention program can be in context. By evaluating the extant literature through the lens of clinical utility and the biopsychosocial model there is a potential to improve medical outcomes and functional ability outcomes to reduce potential disability and have adolescents with T1D live the fullest possible lives across all domains.

## The Present Study

The purpose of this paper was, therefore, to review and evaluate the clinical utility of psychoeducational intervention studies designed to enhance diabetes self-management in youth. The specific objectives of this review were to: (a) Conduct a systematic search of published studies evaluating psychoeducational interventions for adolescents with T1D; (b) map the clinical utility of the identified research based on a range of indicators, including the problem base, context placement, information gain, transparency, pragmatism, and patient-centeredness of the research; and (c) outline future directions and implications for clinical research and practice.

To achieve these goals, a scoping review of the literature was performed. An overarching aim of this work was to improve the reporting and delivery of psychoeducational interventions for youth with T1D so that they may be implemented by healthcare providers, educators, patients, and families to improve patient outcomes.

## Methods

The methodology for this review was based on the framework of Arksey and O’Malley (2005) and recommendations put forth by Levac et al. (2010). A scoping review is an approach to research synthesis that aims to “map the literature on a particular topic […] and provide an opportunity to identify key concepts; gaps in the research; and types and sources of evidence to inform practice, policymaking, and research” (Pham et al., 2014, p. 373). There were five main stages of the review: (a) identifying the research questions, (b) identifying relevant studies, (c) study selection, (d) charting the data, and (e) collating, summarizing, and reporting the results. The search terms, definitions and strategies employed for this review were largely based on those outlined by the authors of previous reviews (e.g., Hampson et al., 2001; Murphy et al., 2006).

### Identifying the Research Questions

The review was guided by the broad question: What is the clinical utility of evaluative psychoeducational intervention studies to improve diabetes management in youth with T1D? Targeted questions largely based on the clinical utility organizational framework were also posed: Is there a health problem important enough to be addressed by the literature (Frazier, 2020)? Has existing evidence and theory been used to contextualize the research in homes, schools, and communities (Abraham et al., 2018)? Is the literature informative (Abraham et al., 2018)? Is the research accessible and verifiable (Chan et al., 2014)? Is the research applicable to real-life circumstances (Clarke et al., 2007)? and Does the research incorporate patients’ perspectives and priorities (Bolton & Gillett, 2019)?.

### Identifying Relevant Studies

Three electronic databases, PsycINFO (1987-), MEDLINE and CINHAL, were searched for research published from January 2005 until October 2020. These databases were selected due to their focus on disciplines of relevance to the topic: psychology, medicine, and nursing. The search strategy was primarily defined by condition, age, intervention, and outcomes. There was slight variation regarding the specific search terms entered into each database depending on the structure of the database employed. However, each search included, at a minimum, the terms *diabetes, adolescent/adolescence*, AND *intervention*. MEDLINE and CINHAL database searches also included terms to define the type of intervention (e.g., *psychological* OR *social* OR *psychosocial* OR *education*). Synonyms for *diabetes, adolescent/adolescence* and *intervention* were also employed. For example, the synonyms used for *adolescent/adolescence* included *teen* OR *youth* OR *child/children* OR *young person/people* OR *puberty*. All terms were searched as a keyword or as text words appearing in the title or abstract. Moreover, all entries were exported into the reference managing system, Zotero, and any duplicate references were removed. Cited reference searches for previous systematic reviews by Hampson et al. (2001) and Murphy et al. (2006) were also performed in Google Scholar.

### Study Selection

#### Initial Screening – Title and Abstract Check

**Figure 1:**
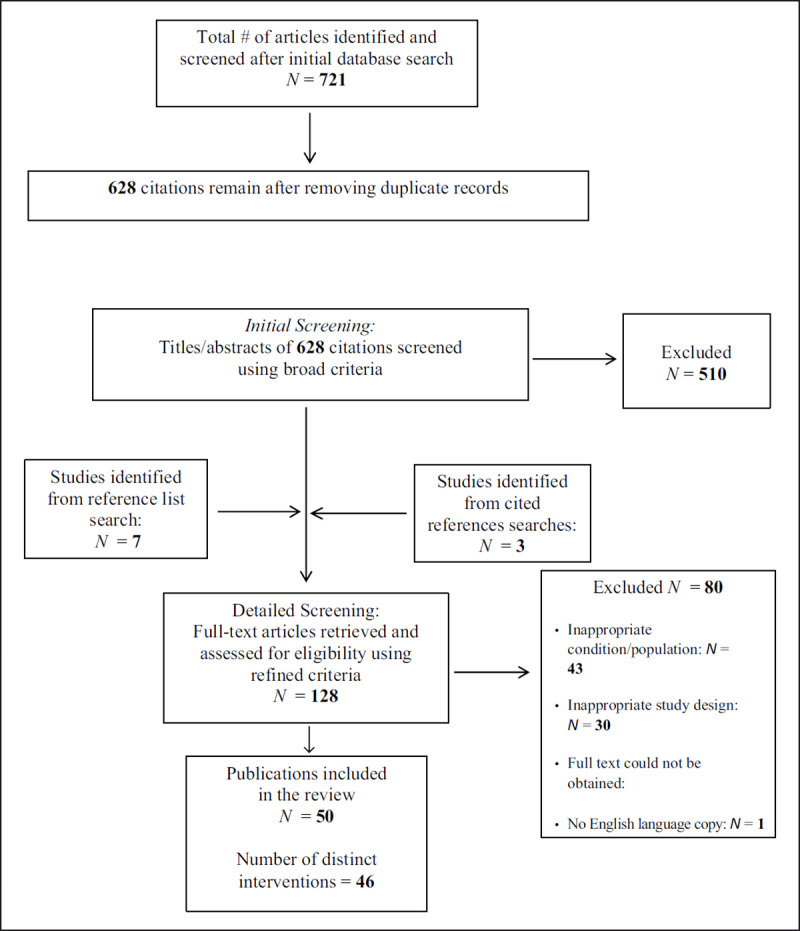
Literature Search Strategies and Decision Tree.

Study selection was performed by one reviewer (LB) in an un-blinded standardized manner. During initial screening, the titles and abstracts of all articles were reviewed using a set of broad criteria as outlined by Hampson et al. (2001). Specifically, articles were retained if (a) the research was primarily about T1D, (b) adolescents were included in the study, and (c) psychoeducational interventions were evaluated. Studies that clearly did not meet these criteria were automatically excluded from further screening. A broad definition of psychoeducational interventions was employed, including interventions designed to change diabetes-related knowledge and behaviour, provide psychosocial training and support, as well as individualized or family-focused counselling. During the initial screening phase, any duplicate references were identified and removed from Zotero. The citations were organized into folders labelled as background literature, excluded articles, or primary studies. Full-text articles for primary studies were retrieved online. See ***Figure 1*** for the workflow of the literature selection.

#### Detailed Screening – Initial Paper Review

After the initial screening, the single reviewer conducted the detailed screening process to decide about including studies in the review. The refined inclusion and exclusion criteria were as follows.

**Inclusion Criteria.** The key criteria for inclusion in this review were that the paper referred to (a) T1D, (b) adolescents aged 12 to 18 years, (c) psychoeducational interventions, and (d) the measurement of intervention effects on participant outcomes.

**Exclusion Criteria.** Papers were excluded for the following reasons: (a) T1D was not the exclusive focus; for example, those focusing on type 2 diabetes or discussing T1D in relation to another health issue (e.g., sexual health) were excluded; (b) the research was not an intervention evaluation; for example, studies discussing the epidemiology of diabetes in a given area or using non-human subjects were excluded; (c) there was no clear investigation of the intervention’s effect on adolescent participants; for example, studies exclusively investigating intervention effects for parents of adolescents with T1D, or people of a wide age range (e.g., 2 to 18 years or 16 to 65 years), were excluded; (d) the research was a small-scale pilot study (*N* < 20), formative evaluation, or employed a purely qualitative design (including case studies); (e) no full-text article was available; or (f) the article was not available in English. ***Figure 1*** depicts the literature search strategies and the number of articles retained and excluded at each stage of the search process.

### Charting the Data

The data from all included studies were compiled in a single data-charting spreadsheet using Microsoft Excel 2013. Information from each study was compiled by a single reviewer. Once all the data were entered, the same reviewer checked the data at random to ensure the information was entered accurately. No authors were contacted for further study information.

### Collating, Summarizing, and Reporting the Results

Upon retrieving the relevant literature, the data were summarized using a narrative and quantitative approach. The concept of clinical utility was defined broadly to describe the usefulness and relevance of psychoeducational interventions studies for clinicians, patients, and families consistent with a biopsychosocial approach. The clinical utility of the literature was evaluated based on six features adapted from Ioannidis (2016): the (a) problem base, (b) context placement (c) information gain, (d) transparency, (e) pragmatism, and (f) patient-centeredness of the research. Frequencies and percentages were calculated to summarize the data and, when possible, effect sizes were computed to consider the magnitude intervention effects across studies. The next sections provide a brief description of how each feature of clinical utility was operationally defined (see also ***Table 1***).

**Table 1:** Summary Data for Selected Indicators of Clinical Utility (n = 50 studies).


FEATURE/INDICATOR	FREQUENCY	PERCENT

Problem Base:		

Reference to disease burden	37	74%

Context Placement:		

Reference to systematic review	28	56%

Use of theory	26	52%

Information Gain:		

Significant reduction in HbA1c (*N* = 41)	15	30%

Adequate power reported (>.80)	21	42%

RCT design	38	76%

Cited > 50 times	14	28%

Transparency:		

Open-access publication	19	38%

Open access to participant-level data	0	0%

Pragmatism:		

Multi-site recruitment (≥2 sites)	27	54%

Sociodemographic information reported	36	72%

Intervention costs reported	5	10%

Patient-Centeredness:		

Medical and psychosocial outcomes	36	72%

Participant satisfaction assessed	21	42%


#### Problem Base

The problem base refers to the burden associated with the medical issue being addressed by the research. As Ioannidis (2016) notes, there is “higher utility in solving problems with higher disease burdens” (p. 2). Thus, it is useful to consider the prevalence, potential health impact, and economic toll of the targeted issue. To evaluate this, each study was examined for references to at least one indicator of the disease burden of T1D.

#### Context Placement

To be clinically useful, new information must also be considered within the context of what is already known (Ioannidis, 2016). Systematic reviews often highlight inconsistencies in research and the need for more studies (Clarke et al., 2007). The use of theory in designing interventions also contextualizes and enhances the utility of clinical research (Corathers et al., 2015). Thus, identified studies were examined for references to recent systematic reviews and the use of a theoretical framework to guide intervention, development, and implementation.

#### Information Gain

The magnitude and quality of the evidence gained from the research are also important indicators of clinical utility. The extent of information gained from the literature was evaluated based on significant changes in patient outcomes associated with the intervention and the magnitude of intervention effects. For clinicians to be confident about the specific benefits of adopting an intervention, sufficiently large intervention effects are required (Ioannidis, 2016). Effect size provides an estimate of the magnitude of the intervention effects, with higher positive numbers indicating larger effects in the anticipated direction. In this review, effect sizes were interpreted based on the values commonly used in behavioural sciences, with <0.20 representing a small effect, 0.50 a medium effect, and >0.80 a large effect.

Given that intervention effects may be obscured by study bias, the quality of the studies was also considered. In particular, studies were evaluated in relation to statistical power, the use of an appropriate control group, the random assignment of participants to groups, the use of an allocation concealment procedure, the steps taken to address missing data, and the use of procedures to ensure the accurate and consistent delivery of interventions.

#### Transparency

The transparency of the research is also worth considering given that it allows for wider dissemination of information. In addition, transparency allows for the identification of major biases in the study design, conduct, and reporting of research, all of which can limit its overall utility (Ioannidis, 2016).

Transparency was evaluated based on the clarity and comprehensiveness of the study methodology, the availability of participant-level data, and whether the research was published in an open-access format. The availability of participant-level data was investigated during the online search process, as well as by searching available entries in clinical trial registries, including the ISRCTN Registry, ClinicalTrials.gov, and the Australian New Zealand Clinical Trials Registry (NZCTR). No authors were contacted to inquire about access to participant data. Open-access publication was confirmed via online searching, the review of journal websites, and through the Directory of Open Access Journals (*https://doaj.org/*).

#### Pragmatism

Pragmatism refers to the extent to which the research is applicable to real-life circumstances (Ioannidis, 2016). The applicability of study results was considered based on participants’ representativeness of the general population of adolescents with T1D. Thus, participant characteristics as well as the number, type, and location of recruitment sources were examined. As well, it can be reasonably assumed that interventions requiring a lot of time and resources are less likely to be adopted by clinicians. Therefore, the identified studies were also examined in relation to these factors.

#### Patient-Centeredness

Useful clinical research is patient-centered; it is well-aligned with patient priorities and deemed acceptable by intervention users (Ioannidis, 2016). As such, included studies were evaluated in relation to the relevance of study outcomes to patients (i.e., use of both medical and psychosocial wellness indicators), and the involvement of intervention users as stakeholders in the research; for example, by assessing participants satisfaction with the intervention.

## Results

### Selection of Intervention Studies

#### Excluded Studies

Electronic database searches yielded a total of 721 results (PsycINFO: 229 results; MEDLINE: 155 results; CINHAL: 337 results), with 628 results remaining after the removal of duplicates. Cited reference searches performed for Hampson et al. (2001) and Murphy et al. (2006) yielded 242 and 125 results, respectively. Using this strategy, 3 new studies were identified (Hampson et al., 2001: 2 results; Murphy et al., 2006: 1 result). In addition, a reference list search performed on a recent comprehensive review of this literature (Bergmame & Shaw, 2018) identified 7 new studies. After initial screening, 128 citations were retained for detailed screening. Following detailed screening, 78 studies were excluded.

#### Included Studies

In total, 50 studies describing 46 distinct interventions were retained for inclusion in the review. The articles described a range of interventions, including technology-driven interventions (13), family-focused interventions (16), as well as individual- and group-format behaviour change interventions (21). Overall, the literature represents a diversity of methods, goals, and outcomes. See ***Table 2***.

**Table 2:** Characteristics of the Identified Studies.


#	CITATION	SAMPLE SIZE, N (I/C)	AGE RANGE	STUDY DESIGN	INTERVENTION(S)	FREQUENCY/DURATION	DELIVERY METHOD	FACILITATOR/SETTING	MAIN RESULTS: BIOMEDICAL/HEALTH BEHAVIOUR	MAIN RESULTS: PSYCHOSOCIAL OUTCOMES

1	Aguilar et al. (2011)	37	9–16 years	Prospective cohort	Education intervention using One Touch UltraSmart	1 session/month over 7 months	Technology	Nurse or physician/medical	Significant average reduction of HbA1c. Significant improvement in dietary habits.	Not assessed.

2	Channon et al. (2007)	66 (38/28)	14–17 years	RCT	Motivational Interviewing (MI)	12 months	Behaviour change (individual)	Health psychologist/ home or community	At 12 months, mean **HbA1c** in the MI group was significantly lower than in the control group after adjusting for baseline values. Difference in HbA1c was maintained at 24 months.	At 12 months, the MI group showed a higher degree of **positive well-being** & improved **quality of life** (i.e., higher life satisfaction, lower life worry, less anxiety, and more positive well-being. There were also differences in **personal models of illness** (e.g., the MI group perceived their diabetes to be more serious and placed greater importance on controlling it).

3	Christie et al. (2014)	362 (181/!81)	8–16 years	RCT	Child and Adolescent Structured Competencies Approach to Diabetes Education (CASCADE): An intensive competency-driven, motivational, psychoeducational program involving patients and families	1 module/month for 4 months	Family -focused	Pediatric specialist nurse/medical	The intervention did not improve HbA1c at 12 months or 24 months.	Intervention group parents at 12 months and adolescents at 24 months had higher scores on **diabetes family responsibility** questionnaire. Adolescents in the intervention group reported **reduced happiness with body weight** at 12 months.

4	Coates et al. (2013)	135 (70/65)	13–19 years	RCT	Carbohydrate, Insulin, Collaborative Education (CHOICE)	Four 3-hour weekly sessions over 1 month	Behaviour change (Individual)	Diabetes specialist nurse and Dietician/community	No significant difference between groups in HbA1c at 12 months; however, there was a significant difference at 24 months. No difference in BMI or in reported hyper or hypoglycemia episodes.	Not assessed.

5	de Wit et al. (2008)	91 (46/45)	13–17 years	RCT	Monitoring and discussing health-related quality of life (HRQoL) with adolescent patients	3 regularly scheduled visits in 12 months	Behaviour change (individual)	Pediatrician/medical	No significant differences between groups over time for **HbA1c** levels.	Means scores on **psychosocial health, behaviour, mental health** and **family activities** improved in the intervention group, except for adolescents with the highest HbA1c. Adolescents in the intervention groups reported higher **self-esteem** at follow-up regardless of HbA1c, and were more **satisfied with care** than control subjects.

6	de Wit et al (2010)	81 (41/40)	13–17 years	RCT	Monitoring and discussing health-related quality of life (HRQoL) with adolescent patients	3 regularly scheduled visits in 12 months *1 year follow-up study	Behaviour change (individual)	Pediatrician/medical	12 months post-intervention, **HbA1c** values had increased significantly.	Mean scores on **behaviour, mental health** and **self-esteem** had significantly decreased, whereas **family activities** subscale remained stable. Adolescents were also less **satisfied with their care**.

7	Ellis et al. (2005)	127 (64/63)	10–17 years	RCT	Multi-Systemic Therapy (MST): Intensive, family-centered and community-based intervention	6 months	Family-focused	Family therapist/home	Participation in MST improved **HbA1c** through **regimen adherence** (mediator).	MST was associated with significant reductions in **diabetes-related stress**.

8	Galler et al. (2020)	31,861 (12,326/19,535)	11–17 years	Record review	Comparison of German adolescents receiving any type of psychological support and those without psychological support	2009–2017	Short-term and continuous care psychology or psychiatry support (individual)	Psyshcologist/variable	Those receiving psychological support were significantly worse in **HbA1c, BMI**, and hospital admission rate. Direction of causality not investigated.	Worse metabolic outcomes associated with need for psychological care and receipt of psychiatric diagnosis.

9	Garcia-Perez et al. (2010)	55 (34/21)	11–18 years	Prospective cohort	Psycho-educative intervention implemented in a summer camp consisting of	8 days	Behaviour change (group)	Diabetes educator and psychologist/summer camp	No significant changes in **HbA1c, BMI, medical visits** or **hospital admission** from pre- to post-intervention.	No significant changes in **diabetes knowledge** or **anxiety** after receiving the intervention.

					medical, educational, and psychosocial components (e.g., interactive seminars about diet, hygiene, recognition and management of hypo- and hyperglycemia, as well as relax seminars and games)					

10	Graue (2005)	101 (55/46)	11–17 years	RCT	Structured educational and counselling program combining group visits and individual computer-assisted consultations	15 months	Technology	Physician, diabetes nurse specialist, dietician, clinical psychologist & social worker/medical	No significant effect on mean **HbA1c**.	Significant age-by-group interactions for **diabetes-related impact, worries, mental health** and **general behaviours**, implying that the intervention was effective for adolescents above 13/14 years of age

11	Grey et al. (2013)	320 (167/153)	11–14 years	RCT	TEENCOPE: Internet-based Coping Skills Training (CST) Managing diabetes: Internet-based diabetes education and problem-solving program (comparison group)	1 session per week over 5 weeks	Technology	Technology/home	At 12 months, there were no significant differences between intervention groups in terms of HbA1c. At 18 months, significantly lower **HbA1c** was noted for youth completing both groups versus just one intervention.	There were no significant differences between intervention groups in relation to **QoL** at 12 months. Youth in both groups had stable QoL (i.e., no change from pre- to post-intervention). At 18 months, higher **QOL, social acceptance** and **self-efficacy**, as well as lower **perceived stress** and **diabetes family conflict** for youth completing both groups.

12	Guo (2020)	100 (50/50)	12–20 years	RCT	Coping skills training contained sessions on goal setting, communication, social	Six 90-minute sessions presented in a 3-day camp setting	Behaviour change (group)	Diabetes educators/summer camp	No change in **HbA1c**, perceived stress, coping style, quality of life.	Feasible and well accepted. However, the program had no effect on any of the dependent variables.

					problem-solving, conflict management, stress management, and positive self-talk					

13	Hilliard et al. (2020)	80 (55/24)	12–17 years	RCT	DoingWell app designed to support parents and reinforce positive teen’s diabetes-related behaviours	Mean of 106 days using the app at least one time per day for 80% of days	Family-focused	Technology/home	No change in HbA1c, resilience, family conflict, self-management, quality of life, or self-case inventory.	Feasible and well accepted. However, the program had no effect on any of the dependent variables.

14	Holmes et al. (2014)	226 families (137/89)	11–14 years	RCT	Coping program: Individualized, intensive family teamwork coping skills training Education program: Psychologically supportive education program to maintain parental involvement and disease care throughout early adolescence	4 quarterly appointments over 1 year	Family-focused	University-affiliated interventionist/medical	Rate of change in **HbA1c** over time was significantly better for the Education versus Usual Care (UC) group, and for the Education versus Coping group (i.e., glycemic control improved in the Education group over time compared with the other two groups). HbA1c of the Coping and UC groups did not differ from one another. Education group improved in **diabetes adherence** across all follow-ups and improved more over time relative to the Coping group. The Coping group demonstrated sustained diabetes adherence.	Both groups showed lower levels of **parental monitoring** over time, although the Education group tended to have more parental monitoring than the Coping group over time. Both groups had **positive parental expectations about involvement**. No significant changes in **diabetes-related** and **general family conflict**, or **self-efficacy**.

15	Husted et al. (2014)	71 (37/34)	13–18 years	RCT	Guided Self-Determination- Youth (GSD-Y): A life skills approach to facilitate empowerment in the patient-provider relationship, adapted for adolescents and their parents	8 sessions over 8–12 months	Behaviour change (individual)	Physicians, nurses, dietician/medical	No significant effect on HbA1c.	GSD-Y significantly reduced the **motivation for diabetes self-managemen**t after adjusting for the baseline value.

16	Iafusco et al. (2011)	396 (193/203)	10–18 years	Prospective cohort	Chat line supervised and moderated by a physician; took place once a week. The topic of each session was chosen and voted on by all participants at the beginning of the chat and might concern diabetes management, as well as anxiety about the future and interpersonal and social relationships (sexual life, travels, etc.)	1 chat per week over 2 years	Technology	Physician/home	Significant decrease in HbA1c in patients who participated in chat session compared with the controls. No difference was observed in HbA1c between the two groups.	Significant improvement **diabetes-related QoL** in patients who participated in chat sessions.

17	Jaser, Patel et al. (2014)	39 (20/19)	13–17 years	Pilot RCT	Check-It!: A positive psychology intervention designed to increase positive affect (PA) through gratitude, self-affirmation, small gifts, and parental affirmations Attentional control (Education) condition: Mailed diabetes educational materials	Every 2 weeks over an 8-week period	Behaviour change (individual)	Program facilitator and parent/home	No main effects for treatment were observed at 6-month follow-up.	A significant association between adolescents’ level of positive affect and measures of adherence (including self-report and metered blood glucose monitoring) was found.

18	Jaser et al. (2014)	320 (167/153)	11–14 years	RCT	TEENCOPE: Internet-based CST Managing Diabetes: Internet based diabetes education and problem-solving program	1 session per week over 5 weeks	Technology	Technology/home	No significant effects of either intervention on **HbA1c**. No significant between-group intervention effects.	Both groups showed significant improvements in **QOL** over time. No significant between-group intervention effects. Self-efficacy mediated the effects on quality of life in both interventions.

19	Kassai et al. (2015)	77 (39/38)	12–17 years	RCT	Nurse counselling intervention	1 pediatrician visit per month, 1 nurse visit and phone calls over 3 months	Behaviour change (individual)	Physician and nurse/medical	The evolution of A1C over the follow-up period was not significantly influenced by the nurse intervention.	Participants’ acceptance of the disease did not change over time.

20	Katz (2014)	153 (50/52/51)	8–16 years	RCT	Care ambassador (CA+) and family-based psychoeducation	30-minute quarterly sessions over 2 years	Family-focused	Research assistant/medical and home	No differences in **HbA1c** across treatment groups. Among youth with suboptimal baseline A1c, more youth in the CA+ psychoeducation group maintained or improved their HbA1c.	Among youth with suboptimal baseline A1c, significant increase in parent involvement in the CA+ psychoeducation than in the other groups (i.e., standard care or CA alone) without negative impact on youth QOL or increased diabetes-specific family conflict.

21	Kichler (2013)	30 (15/15)	13–17 years	RCT	K.I.D.S project: A synthesis of treatment strategies from diabetes education, behaviour therapy, and family therapy; separate group sessions conducted for adolescents and parents	Six 30–45-minute sessions	Family-focused	Psychologist/mental health clinic	No statistically significant changes in **HbA1c** and healthcare utilization from 6 months prior to 6 months post-treatment.	At 4 months post-treatment, parents and youth reported increased **parent responsibility** and parents reported improved youth **diabetes-specific quality of life**.

22	Lawson (2005)	46 (23/23)	13–17 years	RCT	Regular standardized telephone contact with a diabetes nurse educator, including a review of blood glucose results and insulin dose adjustments, problem-solving and diabetes education.	Weekly telephone contact over 6 months	Technology	Diabetes nurse educator/medical – home	Intervention had no immediate effect. However, 6 months post-treatment, **HbA1c** levels decreased in 6 out of 21 individuals of the study group and 0/18 of the control group, while Hba1c increased in 4/21 study subjects and 8/18 control subjects.	Intervention had no effect on **OoL** immediately following or 6 months post-intervention.

23	Maranda (2015)	28 (16/12)	10–17 years	Pilot RCT	Structured care of a betta splendens fish: Participants were instructed to check glucose readings and review glucose logs at times corresponding to the care of the betta fish	3 months	Behaviour change (individual)	Researcher/home	After 3 months, participants in the intervention group showed a significant decrease in **HbA1c** level compared to controls who had an increase. No significant effects on **self-management**.	No significant effects for Pediatric QoL. Younger adolescents (10–13 years) demonstrated a significantly greater response to the intervention than older adolescents (14–17 years).

24	Monaghan et al. (2015)	30 families	11–15 years	Prospective Cohort	Checking In: A physician delivered intervention to increase parent-adolescent communication	12 weeks	Family-focused	Physician/medical	Overall, no significant change in indicators of glycemic control (HbA1c, blood glucose monitoring, mean blood glucose) from pre- to post- intervention. However, participants who reported adhering to the intervention (*n* = 15) demonstrated a significant increase in **BG-monitoring frequency**.	Parent-reported **conflict surrounding diabetes management** significantly decreased from pre- to post-intervention.

25	Mulvaney (2012)	46 (23/23)	13–17 years	Prospective cohort	SuperEgo: Text messaging intervention providing a combination of guidance and choice for users via individually tailored messages	3 months	Technology	Technology/home or community	Mean **HbA1c** remained unchanged in the intervention group, but significantly increased in the control group.	Not assessed.

26	Murphy et al. (2012)	305 (158/147)	11–16 years	RCT	Families and Adolescents Communication and Teamwork Study (FACTS): A family-centred group education program	1 session per month over 6 months.	Family-focused	Health professionals/medical	12 months post-intervention, there was no significant difference in **HbA1c** in either group and no between group differences over time.	Adolescents perceived no changes in **parental input** at 12 months.

27	Murphy et al. (2007)	78 children and adolescents (40/38)	6–11 or 12–16 years	RCT	Families and Adolescents Communication and Teamwork Study (FACTS): A family-centred group education program	4 educational sessions over 1 year	Family-focused	Health professionals/medical	No significant difference in **HbA1c** between participants randomized to the immediate or delayed program (control group). For youth who attended ≥ 2, HbA1c fell by 0.29% compared with an increase in non-attenders.	No significant difference between groups in **parental responsibility**. However, at 12-month follow-up, families who attended two or more sessions reported a significant increase in **parental involvement**.

28	Nansel et al., 2012	390 families (201/189)	9–15 years	RCT	WE-CAN manage diabetes: A clinic-integrated behavioural intervention designed to help families improve diabetes management by facilitating problem-solving skills, communication skills, and appropriate responsibility sharing	24 months	Family-focused	Trained non-professional/medical	Significant overall intervention effect on change in **Hba1c** from baseline to 24-month interval. A significant intervention-by-age interaction; among participants **aged 12 to 14**, a significant effect on **glycemic control** was observed, but there was no effect among those aged 9 to 11. No intervention effect on child or parent report of **adherence**.	Not assessed.

29	Nansel et al. (2015)	136 (66/70)	8–16 years	RCT	Family-based behavioural intervention integrating motivational interviewing, active learning, and applied problem-solving to improve dietary intake of youth with diabetes	12 months	Family-focused	Trained non-professional/medical	No significant difference between groups in **HbA1c** across the study duration. There was a positive intervention effect across the study duration for **diet quality**.	Not assessed.

30	Nansel, Thomas, & Liu (2015)	390 families (201/189)	9–15 years	RCT	WE-CAN manage diabetes: A clinic-integrated behavioural intervention designed to help families improve diabetes management by facilitating problem-solving skills, communication skills, and appropriate responsibility sharing	21 months	Family-focused	Trained non-professional/medical	Significant overall effect of treatment on change in **HbA1c** from baseline to follow-up. Baseline HbA1c was significantly poorer in the low-income group. Interaction for treatment-by-income was not significant.	Not assessed.

31	Nansel et al. (2007)	81 (40/41)	11–16 years	RCT	Diabetes “Personal Trainer” intervention designed to enhance motivation and capability for diabetes management	6 sessions over 2 months	Behaviour change (individual)	Trained non-professional/home or community	At both short-term and 1 year follow-up, there was a significant intervention-by-age interaction, indicating a greater effect on **HbA1c** among older than younger youth; no treatment group differences among pre-/early adolescents (11–13 years), but a significant difference among middle adolescents (14–16 years).	No treatment group differences in parent or youth report of adherence.

32	Nansel et al. (2009)	81 (40/41)	11–16 years	RCT	Diabetes “Personal Trainer” intervention –designed to enhance motivation and capability for diabetes management	6 sessions over 2 months	Behaviour change (individual)	Trained non-professional/home or community	Significant intervention effects on HbA1c among middle adolescents maintained at 1-year follow-up.	Not assessed.

33	Newton & Ashley (2013)	59 (25/25)	13–18 years	RCT	Diabetes Teen Talk: Web-based intervention that provides teens with opportunities to discuss solutions to psychosocial problems that make treatment compliance difficult	7 weeks	Technology	Technology with moderator/home	Not assessed.	Marginally significant difference between groups on combined outcome measures: Diabetes-related QoL, self-efficacy and outcome expectations. Effect of the treatment condition was predominantly carried by a significant difference between treatment conditions on the **Positive Outcomes Expectations** (with those in the control group reported higher outcome expectations).

34	Newton et al. (2009)	78 (38/40)	11–18 years	RCT	Use of an open pedometer & motivational text messages reminding users to wear the pedometer and be active	12 weeks	Technology	Home and community	No significant differences in secondary measures: **HbA1c, blood pressure, BMI**. At 12 weeks, there was no significant difference in change in **physical activity** measures between the groups.	No significant differences in **QoL**.

35	Nicholas et al. (2012)	31 (15/16)	12–17 years	RCT	Online education and support website intervention combining three key components: diabetes-based information, interactive learning activities, and discussion topics relevant to adolescents	8 weeks	Technology	Technology with moderator/home and community	Not assessed.	Pre-post intervention gains approaching significance (at .05 level) in **perceived social support** (i.e., awareness of relationships with others outside of participants’ family).

36	Noyes et al. (2020)	308 (190/103) with significant attrition	6–18 years	RCT	Standardized self-management kits	6 months	Technology	Family with diabetes educator support/medical	Standardised kits showed no evidence of benefit, inhibited diabetes self-management and increased worry.	Information-only kits resulted in no change. Many participants were unwilling to pay the cost of the kits.

37	Price et al. (2016)	396 (199/197)	11–16 years	RCT	KICk–OFF: A group education course designed to meet the learning styles of adolescents. It employs interactive and practical learning activities focusing on carbohydrate counting and insulin adjustment in everyday life	5 days	Behaviour change (group)	Nurse and dietician/community	HbA1c was no different at 24 months.	Significantly improved total QoL scores within 6 months.

38	Ramírez-Mendoza et al. (2020)	121	8 –13 years	Prospective cohort	PANDA: interdisciplinary care plan to empower children in self-management	6 months	Behavior change (group)	Interdisciplinary care plan includes three integrative areas: social work, pediatric nursing and endocrinology	Improvements in HbA1c and glycemic variability.	The program resulted in improvements over time.

39	Serlachius et al. (2016)	147 (73/74)	13–16 years	RCT	Best of Coping (BOC) program: A cognitive behaviour-therapy-based program to improve glycemic control and psychosocial well-being	Five 2-hour long weekly sessions	Behaviour change (group)	Health psychologist/hospital	No difference in **HbA1c** between groups at follow-up.	**Psychosocial well-being** improved in the intervention group compared to the control group.

40	Spiegel et al. (2012)	66 (33/33)	12–18 years	RCT	Nutrition education intervention, which involved attending an educational class offered by a registered dietician/certified diabetes educator and keeping 3-day food records	One 90-minute class, and the completion of 2 sets of 3-day food records	Behaviour change (group)	Dietician and diabetes educator/medical	At 3-month follow-up, the overall intervention effect was not statistically significant for change in **HbA1c** or **carbohydrate counting accuracy**.	Not assessed.

41	Verbeek et al.(2011)	25	11–17 years	Prospective cohort	Psycho-educational intervention focusing on importance of adequate BG monitoring, difficulties to achieving good glycemic control, importance of good diet, and the psychological aspects of coping with diabetes	Four 1.5-hour sessions over 3 months	Behaviour change (group)	Diabetes nurse/medical	HbA1c levels decreased by 0.65 % after 9-month follow-up. A subgroup of 15 patients showed a clinical significant HbA1c reduction at 9-month follow-up with a mean reduction of 1.6 %.	Not assessed.

42	Viklund et al.(2007)	32 (18/14)	12–17 years	RCT	Empowerment education program involving group sessions	Six 2-hour sessions over approximately 6 weeks	Behaviour change (group)		**HbA1c** was similar in the intervention and control group 6 months after the intervention. HbA1c significantly increased among adolescents in the intervention at 6- and 12-month follow-up but returned to baseline levels 18 months after the program.	At 6-month follow-up, there was no difference between the groups in terms of **empowerment**.

43	Von Sengbusch et al. (2005)	104 youth & 95 parents	8–16 years	Prospective cohort	Provision of a mobile diabetes education and care team to families who have limited access to specialized diabetes care in rural areas	1 or 2 educational sessions/week over 2 years	Family-focused	Physician and nurse/medical	**HbA1c** values significantly improved and **rate of hospitalization** fell, from baseline to follow-up.	Youth reported significantly better **diabetes-specific quality of life** and higher **self-esteem** after the intervention. Theoretical **diabetes knowledge** increased at both short- and long-term follow-up.

44	Waller et al. (2008)	48	11–16 years	Prospective cohort	Kids in Control of Food (KICk-OFF): A modular educational program providing information on carbohydrate counting and insulin adjustment	6 courses delivered over 5 school days	Behaviour change (group)	Pediatric diabetes nurse and dietician/school	No changes in **HbA1c, BMI**, or **episodes of hypoglycemia**.	Youths and parents reported significantly improved **QoL** (generic and diabetes-specific) as well as **satisfaction with treatment** at 6-month follow-up. Youth reported improved **self-efficacy**, and both youth and their parents reported greater **child responsibility** for a range of management tasks. No significant changes in either youth- or parent-reported **family conflict**.

45	Wang et al. (2010)	43 (21/22)	12–18 years	RCT	Motivational Interviewing (MI) in Education Structured Diabetes Education (SDE)	2–3 sessions over a 3- to 4-month period	Behaviour change (group)	Diabetes educator/medical	At 6-month follow-up, youth participating in SDE had significantly lower mean **HbA1c** than youths in the MI group.	No between-group differences on any psychosocial measures (i.e., **QoL, stress, self-efficacy, self-perception**, or **family conflict**).

46	Whittemore et al. (2012)	320 (167/153)	11–14 years	RCT	TEENCOPE: Internet-based CST Managing Diabetes: Internet-based diabetes education and problem-solving program	6 months	Technology	Technology/home and community	**HbA1c** significantly increased in the Managing Diabetes group. No significant between-group treatment effects 6 months post-intervention on HbA1c.	At 6 months, no significant between-group treatment effects on **QoL**.

47	Whittemore et al. (2016)	124 (64/60)	11–14 years	RCT	Teens.Connect: Combines Managing Diabetes & TEENCOPE – an interactive Internet program aimed at increasing teens’ coping and social self-efficacy Planet D + discussion board: An open-access diabetes website for youth providing age- appropriate diabetes education	1 session per week over 5 weeks	Technology	Technology/home and community	After 6 months, there were no significant differences in **HbA1c** between groups.	No significant between-group differences in **QoL** or secondary outcomes (i.e., **self-efficacy, self-care, perceived stress, depression**) at 6 months. Teens in the Teens.Connect group reported lower **perceived stress** over time (*p* < 0.01).

48	Wysocki et al. (2006)	104 families (36/36/32)	11–16 years	RCT	Behavioural Family Systems Therapy for Diabetes (BFST-D): A modified BFST intervention to achieve greater impact on diabetes-related family conflict, treatment adherence, and metabolic control Multifamily educational support (ES)	12 sessions over 6 months	Family-focused	Psychologist or social worker/medical	BFST-D and ES significantly improved **HbA1c** compared to standard care **among those with poorer metabolic control** at baseline.	BFST-D significantly improved **family conflict** and **adherence** compared to ES and standard care, especially among those with poorer metabolic control.

49	Wysocki et al. (2007)	104 families (36/36/32)	11–16 years	RCT	Behavioural Family Systems Therapy for Diabetes (BFST-D) Multifamily educational support (ES)	12 sessions over 6 months	Family-focused	Psychologist or social worker/medical	BFST-D was superior to ES and standard care in the effects on **HbA1c**. A significantly higher percentage of BFST-D youth achieved moderate or greater improvement in **treatment adherence** compared with the standard care group at each follow-up and the ES group at 6 and 18 months.	There was a consistent reduction in **family conflict** and improved **adherence**, favoring BFST-D over ES and SC. For these outcomes, there were significant main effects for groups, but the group-by-time interaction effects were not significant.

50	Wysocki et al. (2008)	104 families (36/36/32)	11–16 years	RCT	Behavioural Family Systems Therapy for Diabetes (BFST-D) Multifamily educational support (ES)	12 sessions over 6 months	Family-focused	Psychologist or social worker/medical	Improvement in adolescents’ communication was significantly associated with improvements in **HbA1c** scores at 6 months, as well as improved **adherence** at 6 and 12 months.	BFST-D improved individual **communication of adolescents and mothers**, but not fathers. BFST-D significantly improved **quality of family interaction** compared to ES and standard care. Changes in family communication were associated with changes in family conflict.


### Clinical Utility of Interventions

The sections to follow describe the clinical utility of psychoeducational intervention studies in relation to the six features previously described. Frequencies and percentages for each feature of clinical utility are presented in ***Table 1***.

#### Problem Base

All the identified studies addressed self-management challenges among adolescents with T1D, a medical issue that has a high disease burden. In total, 37 of the identified studies (74%) referred to at least one indicator of disease burden, such as the incidence, prevalence, and potential impact of the disease on physical and psychological health. Although most studies included participants with varying degrees of glycemic control, 13 studies (29%) exclusively involved youth with poorly controlled diabetes, who are at a heightened risk for long-term complications and healthcare use. In a few cases, subgroup analyses were performed to compare the effectiveness of interventions for individuals with varying levels of glycemic control (e.g., deWit et al., 2008; Nansel et al., 2015).

#### Context Placement

Over half of the identified studies were informed by recent empirical evidence and theory. Twenty-eight studies (56%) referred to a recent systematic review of the relevant literature, while 26 studies (52%) described a theoretical basis for the intervention. Behavioural theories were most frequently cited (16), followed by motivational theories (9), family systems theory (8), cognitive-behavioural theories (5), and constructivist learning theory (3). Across studies, the use of theory was largely reflected in the goals of the intervention and selection of study outcomes. For example, some interventions employed behavioural learning theories to promote adolescents’ acquisition of new skills (e.g., Maranda et al., 2015) while others involved the use of motivational theory to promote improved adherence to previously learned treatment regimens (e.g., Channon et al., 2007; Husted et al., 2014).

#### Information Gain

Across studies, intervention effectiveness was evaluated in relation to a range of biomedical, behavioural, and psychosocial outcomes. The most common dependent variable by far was HbA1c, which was used in 47 of the identified studies (94%). In 41 studies, change in HbA1c was assessed from pre- to post-intervention, with 15 studies demonstrating a statistically significant reduction in HbA1c levels, and 26 studies showing no significant difference. Diabetes regimen adherence was also frequently evaluated. For example, self- and parent reported adherence questionnaires were included in 20 studies (44%). Of these studies, only 6 reported improvements from pre- to post-intervention, while 14 studies reported no significant changes. Mixed results were also found for parent- and self-reported psychosocial outcomes. The most common psychosocial outcomes assessed from pre- to post-intervention were adolescents’ quality of life (26), followed by diabetes-related self-efficacy (12), perceived stress (6), and well-being (6). Of the 26 studies assessing pre- to post-intervention changes in quality of life, the majority identified significant positive findings (17). Although effect sizes (i.e., standardized measures of the impact of the interventions such as Cohen’s *d*, odd rations, or Hedges’ *g*) were infrequently reported across studies, effect-size calculations based on available study information indicated mixed results. Hedges’ *g*, which provides a measure of effect size weighted according to the relative size of each sample, was calculated to check effect sizes. Most of the identified interventions had either no effects or small effects for medical and psychosocial outcomes (e.g., Christie et al., 2014; Graue et al., 2005); only a few studies demonstrated moderate effects (e.g., Aguilar et al., 2011; Channon et al., 2007).

In many fields, the interpretation of study findings is limited by low-powered study designs. Twenty-six of the identified studies (58%) reported the use of power calculations to ensure an adequate sample size. Of these, 21 had adequate power (i.e., >.80) while 5 had insufficient power. The remaining 24 studies made no reference to statistical power. For studies not reporting power calculations, sample sizes ranged from 25 to 396 participants. It is possible that a subset of these studies was also underpowered (e.g., those with *N* < 50), which may partially contribute to mixed results.

The use of a control group and randomized assignments to groups are also important features of a well-designed study. Thirty-six of the identified studies (72%) reported the use of control groups matched on at least one variable. Thirty-two of these studies used either a waitlist or no intervention condition as the control group. Only four studies employed active control groups (i.e., involving an alternative intervention), which are typically superior to no intervention conditions. Thirty-seven of the identified studies (74%) reported the random assignment of participants to groups. Of these studies, only 14 reported using a concealed allocation procedure to reducing sampling bias. In total, 35 of the included studies (70%) were randomized controlled trials (RCTs), while two were pilot trials for upcoming full-scale RCTs.

Participant attrition was common in the included studies, with 40 studies (80%) reporting missing data. To minimize the risk of attrition bias, an intention to treat (ITT) analysis is typically recommended as it is a more conservative and less biased estimate of intervention effectiveness (Centre for Reviews and Dissemination, 2009). Of the identified studies, nearly half (22; 44%) reported the use of ITT analyses.

Finally, study findings are obscured when the delivery of interventions is inaccurate or inconsistent. Of the identified studies, 17 (34%) reported fidelity checking procedures to ensure that interventions were carried out as planned.

#### Transparency

Although most included studies provided adequate descriptions of the study methods for publication, few provided enough detail for full study replication. Only one report, by Christie et al. (2014), provided a level of detail that could potentially lend itself to study replication. The study was published in *Health Technology Assessment*, an open-access journal publishing detailed reports of research funded by NIHR Health Technology Assessment program. Although this journal permitted the publication of a detailed research summary, it is likely that, for many other authors, the inclusion of full study details was not possible due to journal-specific publication constraints (i.e., maximum word limits). However, several studies provided details regarding intervention and study characteristics beyond the published report in supplemental online documents. As well, 19 of the studies were registered RCTs, allowing readers to access additional study information on trial registry websites (ClinicalTrials.gov: 9; ISRCTN Registry: 7; ANZCTR: 3). In total, 19 studies (38%) were open-access publications. None of the studies provided direct open access to participant-level data.

#### Pragmatism

The applicability of study findings to a range of adolescents with T1D relies heavily on the representativeness of the study sample. The identified studies were conducted in 14 different countries, with the majority completed in the United States (USA; 24), followed by the United Kingdom (UK; 9), Netherlands (4), Canada (2), Spain (2), Norway (1), Denmark (1), Italy (1), France (1), Sweden (1), Mexico (1), Germany (1), Australia (1), and New Zealand (1). For nearly all studies, participant recruitment took place in medical contexts, including pediatric hospitals and diabetes outpatient clinics (44; 98%). Two studies recruited participants from diabetes summer camps. Approximately half of the identified studies (54%) recruited participants from more than one site, while the remaining studies recruited individuals from a single hospital or center. The largest number of recruitment sites within a single study was 31 (Price et al., 2016); most studies involved between one and five sites (39; 78%).

Moreover, 32 studies (64%) reported at least one sociodemographic characteristic of the sample (e.g., race, ethnicity, household income). Out of 27 studies specifying the race or ethnicity of study participants, 26 involved samples consisting of predominantly white, non-minority participants, while one study consisted of predominantly Black participants (Ellis et al., 2005). Furthermore, 12 studies (24%) exclusively involved adolescents with poorly controlled diabetes who may not be representative of all adolescents with T1D.

The usefulness of interventions can also be considered in relation to the amount of time and resources required for implementation. Only five of the studies (10%) reported estimated costs for the delivery of interventions. Two of these studies were UK-based and evaluated intensive multi-component behaviour change interventions (Christie et al., 2014; Waller et al., 2008). The estimated costs for the interventions ranged from £600 to £700 per child, which is equivalent to approximately $745 to 870 USD (based on an exchange rate of 1.2418). A third study was conducted in the USA and evaluated a modified behavioural family systems therapy intervention for adolescents with poorly controlled diabetes (Wysocki et al., 2008). The total estimated cost for this intervention was $2,700 (USD) per family. Overall, the most resource-intensive interventions were those employing a combination of treatment methods and requiring extensive personnel training (e.g., Christie et al., 2014; Wysocki et al., 2006), while the most efficient and cost-effective interventions were those delivered via accessible technologies such as a personal computer or Smartphone (e.g., Grey et al., 2013; Jaser et al., 2014).

The cost-effectiveness of interventions can also be evaluated in terms of patient healthcare use (e.g., hospital admissions); however, only three (6%) of the identified studies included this as an outcome variable (Christie et al., 2014; Kichler et al., 2013; Von Sengbusch et al., 2006). Of these, only one study found a significant decline in the rate of participant hospitalization following the intervention (Von Sengbusch et al., 2006).

#### Patient-Centeredness

Patient-centered research employs outcome measures that are meaningful and relevant to participants. Of the identified studies, 36 (72%) examined both medical and psychosocial outcomes, while 9 (18%) included only medical or adherence-related outcomes, and 3 (6%) included only psychosocial outcomes. Patient-centered research also strives to involve participants as engaged stakeholders in the research. Of the identified studies, 21 (42%) assessed patient satisfaction with the intervention.

Patient feedback was occasionally obtained during the implementation process; for example, when determining the location of interventions sessions. The interventions took place in a range of locations, including medical contexts (26), and patients’ homes or other community settings (19). Although many interventions took place at predetermined times and locations outside of standard medical care (e.g., Coates et al., 2013), some were incorporated into regularly scheduled clinic visits (e.g., de Wit et al., 2008), or delivered more flexibly at locations identified by the patients (e.g., schools and parks; Price et al., 2016).

## Discussion

As illustrated, the literature describing psychoeducational interventions to improve adolescent diabetes management is diverse. A primary goal of this work is to identify effective strategies that can be implemented in real-world contexts to improve adolescents’ health and well-being. However, many factors may impede the translation of clinical research into practice. Previous systematic reviews of the literature have focused almost exclusively on one or two indicators of clinical utility, including study quality and the magnitude of intervention effects. Application of the biopsychosocial approach requires an expanded range of complementary sources of evidence that contribute to establishing the clinical utility of the literature, including consideration of the problem base, context placement, information gain, transparency, pragmatism, and patient-centeredness of the research (Ioannidis, 2016).

All the identified studies can be considered clinically relevant to the extent that they address a potentially life-threatening disease that affects many individuals. The disease burden associated with diabetes is tremendous. T1D is recognized as one of the largest global health crises of this century, with high blood glucose ranked as one of the highest risk factors for premature mortality (World Health Organization, 2016). Further, globally, the incidence of T1D is estimated to be rising by 3% each year, and the economic toll is high (Taddeo et al., 2008). In the USA alone, nearly 14% of the total health budget is spent on diabetes (International Diabetes Federation, 2015).

T1D is frequently diagnosed between the ages of 8 and 12 years, and is the predominant form of disease affecting adolescents (Hampson et al., 2001). Following prescribed diabetes management tasks is essential for the maintenance of physical health and well-being. However, many adolescents struggle to achieve adequate adherence. Without effective interventions, this may lead to a range of serious health and psychosocial challenges as these youth progress into adulthood. The common focus on HbA1c may not be the most relevant outcome variable for interventions, especially in a biopsychosocial model of clinical utility (Chehregosha et al., 2019). Thus, the identification of effective interventions to improve diabetes self-management among youth is of important clinical value. Without such efforts, the economic and health-related impact of T1D will likely continue to rise.

The clinical utility of the literature in relation to each of the remaining features was highly variable. For example, the problem base, context placement, information gain, transparency, pragmatism, and patient-centeredness of the research was uneven in coverage. Many of the studies were well informed by recent empirical evidence and theory. Over half of the studies referenced a recent systematic review and over half employed a theoretical model. Framing the research in a clinical utility model adds clarity and meaning to the work, helping clinicians make more informed decisions in their selection of interventions. The use of theory to guide intervention development is also valuable, as it provides a broad framework from which the intervention may be modified by clinicians to meet the needs of diverse patients (Chilton & Pires-Yfantouda, 2015).

The degree of information gained from each study was mixed in relation to a range of indicators. Specifically, across studies, the measurable effects of the interventions were modest at best for medical and psychosocial outcomes. This is consistent with previously published systematic reviews of the psychoeducational intervention literature (e.g., Hampson et al., 2001; Murphy et al., 2006). Significant positive results were most frequently noted in relation to self-reported quality of life. However, the effectiveness of psychoeducational interventions for promoting improved adherence behaviours and glycemic control (HbA1c) remains unclear. Mixed results could, in part, be attributable to methodological difficulties present in the literature, including insufficient power, use of inadequate control groups, inadequate participant randomization, failure to investigate intervention effectiveness across differing adolescent subgroups (e.g., teens of different ages or baseline HbA1c), as well as the use of questionable outcome measures.

Although improvements in HbA1c are meaningful, use of this outcome measure alone may not be acceptable for clinical decision-making (Lipska & Krumholz, 2017). In this review, most identified studies used HbA1c as a primary outcome measure. The use of HbA1c as an outcome measure has been criticized given that it is affected by a range of factors beyond diabetes regimen compliance (Lipska & Krumholz, 2017), such as hormonal fluctuations (including those associated with puberty), stress levels, pain, illness, and dehydration, as well as the use of medications (e.g., steroids or antipsychotics; American Diabetes Association, 2017).

Despite some methodological shortcomings in the literature, multiple high-quality studies were identified (e.g., Channon et al., 2007; Whittemore et al., 2012). Of the behaviour change interventions reviewed, those employing motivational interviewing demonstrated significant positive effects on health-related and psychosocial outcomes, although these effects were modest at best (Channon et al., 2007; Christie et al., 2014). Family-focused interventions also demonstrated small but positive effects, especially in relation to self-reported quality of life and well-being (Ellis et al., 2005; Kichler et al., 2013). The effectiveness of technology-driven interventions was also mixed; however, a few small to moderate effects were noted (e.g., Aguilar et al., 2011; Jaser et al., 2014).

Though promising results have been identified, questions remain about the applicability of findings to larger, more diverse groups of adolescents with T1D. The identified studies were conducted in more than 10 countries, and only about half of the studies recruited participants from more than one site. Thus, it is important to consider study findings in context. Many of the included samples were homogenous, consisting of predominantly white, non-minority youths. Thus, study results may have limited utility for clinicians working with adolescents of ethnic minority or low socioeconomic backgrounds. This is worth careful consideration given that the incidence of T1D is growing disproportionately in these populations (Kassai et al., 2015).

The identified studies also varied regarding intervention feasibility and cost effectiveness. Like the findings of Hampson et al. (2001), few studies estimated costs for the interventions, which limits the ability to make comparisons across studies. However, it can be reasonably assumed that intensive, multi-component interventions are likely to require more resources and may be more challenging to implement; the delivery of interventions via commonly used technologies, on the other hand, are likely to be more cost-effective and user-friendly. To maximize utility, effective interventions must be delivered in an efficient and cost-effective manner. Thus, future research should investigate ways that interventions can be modified to improve efficiency and reduce costs, while maintaining or increasing the benefits for patients.

Research transparency is another critical variable for the application of research in real-world settings. Clear and detailed reporting can facilitate study replication and ensure that research results are reliable, valid, and applicable to real-life situations. Although the identified studies provided adequate methodological details for publication, only one provided enough detail and clarity to facilitate full study replication. Thus, there are significant gaps in the published literature regarding precise study procedures and the content of the evaluated interventions. Responsible data-sharing can also facilitate more efficient research, help expand the knowledge base, and ultimately, improve patient outcomes (Tudur-Smith et al., 2015). However, none of the identified studies provided direct open access to participant-level data. In addition, open-access publication can substantially broaden the readership and use of an article in clinical settings, yet less than half of the identified studies were published in an open access format.

Thus, increased steps must be taken to ensure that psychoeducational intervention studies and information are accessible to clinicians. This can be accomplished in a variety of ways, for example, through increased dissemination of full study reports and protocols, increased use of preprints, the provision of data-sharing incentives (e.g., data authorship), the inclusion of participant-level data as a condition for publication, increased support for open access publishing, and other components consistent with knowledge translation (Bierer et al., 2017; Chan et al., 2014).

The patient-centeredness of the literature also varied. Although most studies included outcomes of relevance to patients, less than half assessed participants’ level of satisfaction with the interventions. In addition, when participant feedback was obtained, it was often unclear if, and how, this information would be subsequently used to improve interventions. Inviting adolescent involvement in the development and evaluation of interventions can help to ensure the creation and dissemination of research that is relevant to real-life situations and be consistent with the biopsychosocial approach (Ioannidis, 2016). This should, therefore, be emphasized in future research.

Although no single study encompassed all the features of clinical utility listed here, each study had unique strengths and limitations that may facilitate or impede application to routine clinical care. For example, Murphy et al. (2007) evaluated a family-centered group education program consisting of four 1-hour educational sessions delivered over 12 months. The study demonstrated a high degree of patient-centeredness, including meaningful outcomes, assessing an intervention that was integrated into routine clinical care, and incorporating patient feedback during and after the intervention. However, widespread use of the intervention may be difficult given that it requires increased time and resources from healthcare providers who are already busy. As well, at 12-month follow-up, no significant differences between the intervention and control groups were found regarding adolescent HbA1c. Participant attrition resulted in reduced power and differences between study groups, making firm conclusions difficult to draw from the research.

### Implications for Future Research and Clinical Practice

This systematic investigation of psychoeducational interventions for youth with T1D has identified several gaps and shortcomings in the literature to be addressed in future research. Although promising results were found, additional high-quality studies are needed to improve the clinical utility of the literature. One shortcoming of the identified literature is its limited generalizability to the diverse range of adolescents commonly seen in clinical practice. This is a common limitation of clinical research, at least in part, due to challenges associated with participant recruitment and attrition (Lesko et al., 2010). However, continuous efforts should be made to recruit larger and more diverse samples. As well, further research can be conducted to determine whether interventions should be more targeted. For example, the recruitment of larger samples could facilitate subgroup analyses to assess the relative effectiveness of interventions for adolescents of varying ages (e.g., younger vs. older teens), backgrounds, levels of glycemic control, and management challenges (e.g., poor diet vs. infrequent blood glucose monitoring).

The success of an intervention is largely dependent upon the interplay between patient, context, and biopsychosocial factors (Lesko et al., 2010). Thus, in addition to demonstrating intervention effectiveness for a given sample, further research assessing the feasibility of interventions in real-life contexts would be beneficial. Further, consideration of intervention utility should include an estimate of the relative costs to benefits of the intervention. If an intervention is resource-intense yet effective, it may be worthwhile to implement it, given that the costs will eventually be balanced out by a reduced burden on the healthcare system. To date, however, the cumulative costs and long-term consequences of psychoeducational interventions for T1D are largely unknown.

Performing RCTs for all possible clinical questions is likely to be costly and time-consuming; therefore, the use of alternative credible study designs, including methodologically robust (“real-time”) observational studies, should also be considered. Such methodologies can provide additional evidence regarding intervention utility, helping to speed the translation of research into practice (Lesko et al., 2010).

### Limitations of the Review

Although the present review provides new insights and understanding into the clinical utility of psychoeducational intervention studies for adolescents with T1D, it is not without limitations. For practical reasons, the review was limited to studies published within the past 15 years and did not include smaller descriptive studies. Prior to 2008, nearly all published papers included descriptions of programs without significant outcome data or reported on low-powered studies, including pilot studies. A growth of quality studies that evaluated a variety methods and outcomes occurred after the review by Murphy and colleagues (2006).

Unpublished grey literature and non-English studies were also not considered for pragmatic reasons. Publication and language biases can occur when the publication of research is influenced by the results; for example, when studies with significant results are more likely to be published in English language journals as opposed to those with non-significant results (Centre for Reviews and Dissemination, 2008). As such, the inclusion of only published English language studies may have led to an overestimation of intervention effects and overall clinical utility of the literature. Thus, future reviewers should consider extending this inquiry to include a wider range of studies.

A final limitation of this review reflects challenges associated with defining the main features of clinical utility, as well as determining what constitutes individual features like *pragmatic* and *patient-centered*. The criteria employed in this review were not exhaustive. To the author’s knowledge, no set of detailed criteria for evaluating the clinical utility of interventions has been previously published or employed in a systematic manner. In addition, a single reviewer of the clinical literature may have introduced biases into the selection and analysis of studies. Future reviewers examining the clinical utility of medical and psychosocial interventions may wish to refine and adapt the criteria to facilitate further understanding of this topic.

## Conclusions

A primary objective of this review was to map the clinical utility of the literature describing psychoeducational interventions designed to improve medical management of T1D in adolescence. In doing so, several gaps and shortcomings of the literature were identified. Although the extant literature provides clinicians, patients, and families with a range of psychoeducational intervention options to choose from, the clinical utility of these interventions is highly variable. Thus, the selection and implementation of interventions in clinical settings will likely remain a challenge until further research is conducted. Given this reality, the implication for clinical researchers is to conduct high-quality studies that provide strong evidence for the application of findings to real-world settings.

Ioannidis’ framework (2016) concerning clinical utility describes factors that are required for a true biopsychosocial model to be implemented. Future research opportunities include components of open science and implementation science in addition to considerations of clinical utility to improve research-to-practice. These components include open access to papers, shared data, transparent methods and instruments, analysis of barriers to implementation, cost analysis, and cultural and systemic factors. When couched in these frameworks, the literature can better inform clinical practices, as researchers can ensure that their work is well informed by existing evidence and theory, sufficiently informative, accessible and verifiable, applicable to real-life circumstances, and well aligned with patient priorities (Ioannidis, 2016). With greater emphasis on each of these features, the literature will have farther-reaching clinical implications with a biopsychosocial approach and, ideally, help to reduce the burden of T1D on adolescents, their families, and the healthcare system.

## References

[1] Abraham, M. B., Jones, T. W., Naranjo, D., Karges, B., Oduwole, A., Tauschmann, M., & Maahs, D. M. (2018). ISPAD clinical practice consensus guidelines 2018: Assessment and management of hypoglycemia in children and adolescents with diabetes. Pediatric Diabetes, 19, 178–192. DOI: 10.1111/pedi.12698 29869358

[2] Aguilar, M. J., Pedro, A. G., Gonzalez, E., Perez, M. C., & Padilla, C. A. (2011). A nursing educational intervention helped by One Touch UltraSmartTM improves monitoring and glycated haemoglobin levels in type I diabetic children. Journal of Clinical Nursing, 21, 1024–1032. DOI: 10.1111/j.1365-2702.2011.03926.x 22221300

[3] Akhter, K., Turnbull, T., & Simmons, D. (2018). A systematic review of parent/peer-based group interventions for adolescents with type 1 diabetes: Interventions based on theoretical/therapeutic frameworks. British Journal of Diabetes, 18(2), 51–65. DOI: 10.15277/bjd.2018.177

[4] American Diabetes Association. (2017). Factors affecting blood glucose. Retrieved from http://www.diabetes.org/living-with-diabetes/treatment-and-care/blood-glucose-control/factors-affecting-blood-glucose.html

[5] Arksey, H., & O’Malley, L. (2005). Scoping studies: Towards a methodological framework. International Journal of Social Research Methodology, 8(1), 19–32. DOI: 10.1080/1364557032000119616

[6] Bergmame, L., & Shaw, S. (2018). Psychoeducational interventions to improve adolescents’ medical management of diabetes: A comprehensive review. Health Psychology Report, 6(1), 10–39. DOI: 10.5114/hpr.2018.70357

[7] Bierer, B. E., Crosas, M., & Pierce, H. H. (2017). Data authorship as an incentive to data sharing. The New England Journal of Medicine. DOI: 10.1056/NEJMsb1616595 28745980

[8] Bolton, D., & Gillett, G. (2019). The biopsychosocial model of health and disease: New philosophical and scientific developments. Springer Nature. DOI: 10.1007/978-3-030-11899-0 31886965

[9] Caccavale, L. J., & Monaghan, M. (2020). Behavioral interventions for youth with diabetes. Journal of Health Service Psychology, 46(3), 109–117. DOI: 10.1007/s42843-020-00014-1

[10] Canadian Diabetes Association. (2013). Canadian diabetes association 2013 clinical practice guidelines for the prevention and management of diabetes in Canada. Canadian Journal of Diabetes, 37(supplement 1), S1–S212. DOI: 10.1016/j.jcjd.2013.02.056 24070926

[11] Carroll, N. C., & Vittrup, B. (2020). Type 1 diabetes in adolescence: considerations for mental health professionals. Journal of Child and Adolescent Counseling, 6(2), 137–148. DOI: 10.1080/23727810.2020.1729010

[12] Centre for Reviews and Dissemination. (2008). Systematic reviews: CRD’s guidance for undertaking reviews in health care. CRD, University of York.

[13] Chan, A-W., Song, F., Vickers, A., Jefferson, T., Dickersin, K., Gøtzsche, P. C., …van der Worp, B. H. (2014). Increasing value and reducing waste: Addressing inaccessible research. The Lancet, 383(9913), 257–266. DOI: 10.1016/S0140-6736(13)62296-5 PMC4533904 24411650

[14] Channon, S. J., Huws-Thomas, M. V., Rollnick, S., Hood, K., Cannings-John, R. L., Rogers, C., & Gregory, J. W. (2007). A multicenter randomized controlled trial of motivational interviewing in teenagers with diabetes. Diabetes Care, 30(6), 1390–1395. DOI: 10.2337/dc06-2260 17351283

[15] Chehregosha, H., Khamseh, M. E., Malek, M., Hosseinpanah, F., & Ismail-Beigi, F. (2019). A view beyond hba1c: role of continuous glucose monitoring. Diabetes Therapy, 10(3), 853–863. DOI: 10.1007/s13300-019-0619-1 31037553 PMC6531520

[16] Chilton, R., & Pires-Yfantouda, R. (2015). Understanding adolescent type 1 diabetes self-management as an adaptive process: A grounded theory approach. Psychology & Health, 30(12), 1486–1504. DOI: 10.1080/08870446.2015.1062482 26084198

[17] Christie, D., Thompson, R., Sawtell, M., Allen, E., Cairns, J., Smith, F., …Viner, R. (2014). Structured, intensive education maximising engagement, motivation and long-term change for children and young people with diabetes: A cluster randomised controlled trial with integral process and economic evaluation – the CASCADE study. Health Technology Assessment, 18(20), 1–202. DOI: 10.3310/hta18200 PMC4781436 24690402

[18] Clarke, M., Hopewell, S., & Chalmers, I. (2007). Reports of clinical trials should begin and end with up-to-date systematic reviews of other relevant evidence: A status report. Journal of the Royal Society of Medicine, 100, 187190. DOI: 10.1177/014107680710011415 PMC1847744 17404342

[19] Coates, V., Chaney, D., Bunting, B., Shorter, G. W., Shevlin, M., McDougall, A., & Long, A. (2013). Evaluation of the effectiveness of a structured diabetes education programme (CHOICE) on clinical outcomes for adolescents with type 1 diabetes: A randomised controlled trial. Journal of Diabetes & Metabolism, 4(6), 280–286. DOI: 10.4172/2155-6156.1000280

[20] Corathers, S. D., Schoettker, P. J., Clements, M. A., List, B. A., Mullen, D., Ohmer, A., … Lee, J. (2015). Health-system-based interventions to improve care in pediatric and adolescent type 1 diabetes. Current Diabetes Reports, 15(11), 1–11. DOI: 10.1007/s11892-015-0664-8 26374568

[21] de Wit, M., Delemarre-van de Waal, H. A., Bokma, J. A., Haasnoot, K., Houdijk, M. C., Gemke, R. J., & Snoek, F. J. (2008). Monitoring and discussing health-related quality of life in adolescents with type 1 diabetes improve psychosocial well-being: A randomized controlled trial. Diabetes Care, 31(8), 1521–1526. DOI: 10.2337/dc08-0394 18509204 PMC2494630

[22] de Wit, M., Delemarre-van de Waal, H. A., Bokma, J. A., Haasnoot, K., Houdijk, M. C., Gemke, R. J., & Snoek, F. J. (2010). Follow-up results on monitoring and discussing health-related quality of life in adolescent diabetes care: Benefits do not sustain in routine practice. Pediatric Diabetes, 11(3), 175–181. DOI: 10.1111/j.1399-5448.2009.00542.x 19538516

[23] Ellis, D. A., Frey, M. A., Naar-King, S., Templin, T., Cunningham, P. B., & Cakan, N. (2005). The effects of multisystemic therapy on diabetes stress among adolescents with chronically poorly controlled type 1 diabetes: Findings from a randomized, controlled trial. Pediatrics, 116(6), e826–e832. DOI: 10.1542/peds.2005-0638 16322140

[24] Frazier, L. D. (2020). The past, present, and future of the biopsychosocial model: A review of The Biopsychosocial Model of Health and Disease: New philosophical and scientific developments by Derek Bolton and Grant Gillett. New Ideas in Psychology, 57, 100755. DOI: 10.1016/j.newideapsych.2019.100755

[25] Galler, A., Hilgard, D., Bollow, E., Hermann, T., Kretschmer, N., Maier, B., Mönkemöller, K., Schiel, R., & Holl, R. W. (2020). Psychological care in children and adolescents with type 1 diabetes in a real-world setting and associations with metabolic control. Pediatric Diabetes, 21(6), 1050–1058. DOI: 10.1111/pedi.13065 32506592

[26] Garcia-Perez, L., Perestelo-Perez, L., Serrano-Aguilar, P., & Del Mar Trujillo-Martin, M. (2010). Effectiveness of a psychoeducative intervention in a summer camp for children with type 1 diabetes mellitus. The Diabetes Educator, 36(2), 310–317. DOI: 10.1177/0145721710361784 20185607

[27] Graue, M., Wentzel-Larsen, T., Hanestad, B. R., & Sovik, O. (2005). Evaluation of a programme of group visits and computer-assisted consultations in the treatment of adolescents with type 1 diabetes. Diabetic Medicine: A Journal of the British Diabetic Association, 22(11), 1522–1529. DOI: 10.1111/j.1464-5491.2005.01689.x 16241917

[28] Grey, M., Whittemore, R., Jeon, S., Murphy, K., Faulkner, M. S., & Delamater, A. (2013). Internet psycho-education programs improve outcomes in youth with type 1 diabetes. Diabetes Care, 36(9), 2475–2482. DOI: 10.2337/dc12-2199 23579179 PMC3747907

[29] Grey, M., Whittemore, R., & Tamborlane, W. (2002). Depression in type 1 diabetes in children: Natural history and correlates. Journal of Psychosomatic Research, 53, 907–911. DOI: 10.1016/S0022-3999(02)00312-4 12377302

[30] Guo, J., Luo, J., Yang, J., Huang, L., Wiley, J., Liu, F., Li, X., Zhou, Z., & Whittemore, R. (2020). School-aged children with type 1 diabetes benefit more from a coping skills training program than adolescents in China: 12-month outcomes of a randomized clinical trial. Pediatric Diabetes, 21(3), 524–532. DOI: 10.1111/pedi.12975 31885120

[31] Hampson, S. E., Skinner, T. C., Hart, J., Storey, L., Gage, H., Foxcroft, D., …Walker, J. (2001). Effect of educational and psychosocial interventions for adolescents with diabetes mellitus: A systematic review. Health Psychology Assessment, 5(10), 1–79. DOI: 10.3310/hta5100 11319990

[32] Hanna, K. M., & Guthrie, D. (2003). Adolescents’ behavioral autonomy related to diabetes management and adolescent activities/rules. The Diabetes Educator, 29(2), 283–291. DOI: 10.1177/014572170302900219 12728755

[33] Heinrich, E., Schaper, N. C., & de Vries, N. (2010). Self-management interventions for type 2 diabetes: A systematic review. European Diabetes Nursing, 7(2), 71–76. DOI: 10.1002/edn.160

[34] Hendrieckx, C., Gonder-Frederick, L., Heller, S. R., Snoek, F. J., & Speight, J. (2020). How has psycho-behavioural research advanced our understanding of hypoglycaemia in type 1 diabetes? Diabetic Medicine, 37(3), 409–417. DOI: 10.1111/dme.14205 31814151

[35] Hermanns, N., Ehrmann, D., Finke-Groene, K., & Kulzer, B. (2020). Trends in diabetes self-management education: Where are we coming from and where are we going? A narrative review. Diabetic Medicine, 37(3), 436–447. DOI: 10.1111/dme.14256 32017188

[36] Herzer, M., & Hood, K. K. (2010). Anxiety symptoms in adolescents with type 1 diabetes: Association with blood glucose monitoring and glycemic control. Journal of Pediatric Psychology, 35(4), 415–425. DOI: 10.1093/jpepsy/jsp063 19684117 PMC2858435

[37] Hilliard, M. E., Cao, V. T., Eshtehardi, S. S., Minard, C. G., Saber, R., Thompson, D., Karaviti, L. P., & Anderson, B. J. (2020). Type 1 Doing Well: Pilot feasibility and acceptability study of a strengths-based mhealth app for parents of adolescents with type 1 diabetes. Diabetes Technology & Therapeutics. DOI: 10.1089/dia.2020.0048 PMC7698853 32379496

[38] Holmes, C. S., Chen, R., Mackey, E., Grey, M., & Streisand, R. (2014). Randomized clinical trial of clinic-integrated, low-intensity treatment to prevent deterioration of disease care in adolescents with type 1 diabetes. Diabetes Care, 37(6), 1535–1543. DOI: 10.2337/dc13-1053 24623027 PMC4030089

[39] Husted, G. R., Thorsteinsson, B., Esbensen, B. A., Gluud, C., Winkel, P., Hommel, E., & Zoffmann, V. (2014). Effect of guided self-determination youth intervention integrated into outpatient visits versus treatment as usual on glycemic control and life skills: A randomized clinical trial in adolescents with type 1 diabetes. Trials, 15, 321–332. DOI: 10.1186/1745-6215-15-321 25118146 PMC4247629

[40] Iafusco, D., Galderisi, A., Nocerino, I., Cocca, A., Zuccotti, G., Prisco, F., & Scaramuzza, A. (2011). Chat line for adolescents with type 1 diabetes: A useful tool to improve coping with diabetes: A 2-year follow up study. Diabetes Technology & Therapeutics, 13(5), 551–555. DOI: 10.1089/dia.2010.0188 21406010

[41] International Diabetes Federation. (2015). IDF diabetes atlas – Seventh edition. International Diabetes Federation. Retrieved from http://www.diabetesatlas.org/

[42] Ioannidis, J. P. A. (2016). Why most clinical research is not useful. PLOS Med, 13(6), e1002049. DOI: 10.1371/journal.pmed.1002049 27328301 PMC4915619

[43] Jaser, S. S., Patel, N., Rothman, R. L., Choi, L., & Whittemore, R. (2014). Check it! A randomized pilot of a positive psychology intervention to improve adherence in adolescents with type 1 diabetes. Diabetes Educator, 40(5), 659–667. DOI: 10.1177/0145721714535990 24867917 PMC4283584

[44] Jaser, S. S., Whittemore, R., Chao, A., Jeon, S., Faulkner, M. S., & Grey, M. (2014). Mediators of 12-month outcomes of two internet interventions for youth with type 1 diabetes. Journal of Pediatric Psychology, 39(3), 306–315. DOI: 10.1093/jpepsy/jst081 24163439 PMC3959262

[45] Kassai, B., Rabilloud, M., Bernoux, D., Michal, C., Riche, B., Ginhoux, T., … Nicolino, M. (2015). Management of adolescents with very poorly controlled type 1 diabetes by nurses: A parallel group randomized controlled trial. Trials, 16, 399–405. DOI: 10.1186/s13063-015-0923-7 26350209 PMC4563922

[46] Katz, M. L., Volkening, L. K., Butler, D. A., Anderson, B. J., & Laffel, L. M. (2014). Family-based psychoeducation and care ambassador intervention to improve glycemic control in youth with type 1 diabetes: A randomized trial. Pediatric Diabetes, 15(2), 142–150. DOI: 10.1111/pedi.12065 23914987 PMC3915039

[47] Kichler, J. C., Kaugars, A. S., Marik, P., Nabors, L., & Alemzadeh, R. (2013). Effectiveness of groups for adolescents with type 1 diabetes mellitus and their parents. Families, Systems & Health: The Journal of Collaborative Family Healthcare, 31(3), 280–293. DOI: 10.1037/a0033039 PMC3979844 23957874

[48] Lawson, M. L., Cohen, N., Richardson, C., Orrbine, E., & Pham, B. (2005). A randomized trial of regular standardized telephone contact by a diabetes nurse educator in adolescents with poor diabetes control. Pediatric Diabetes, 6(1), 32–40. DOI: 10.1111/j.1399-543X.2005.00091.x 15787899

[49] Lesko, L. J., Zineh, I., & Huang, S.-M. (2010). What is clinical utility and why should we care? Clinical Pharmacology and Therapeutics, 88(6), 729–733. DOI: 10.1038/clpt.2010.229 21081937

[50] Levac, D., Colquhoun, H., & O’Brien, K. K. (2010). Scoping studies: Advancing the methodology. Implementation Science, 5(69). DOI: 10.1186/1748-5908-5-69 PMC2954944 20854677

[51] Lipska, K. J., & Krumholz, H. M. (2017). Is hemoglobin A1c the right outcome for studies of diabetes? Journal of the American Medical Association, 317(10), 1017–1018. DOI: 10.1001/jama.2017.0029 28125758 PMC5350060

[52] Maranda, L., Lau, M., Stewart, S. M., & Gupta, O. T. (2015). A novel behavioral intervention in adolescents with type 1 diabetes mellitus improves glycemic control: Preliminary results from a pilot randomized control trial. The Diabetes Educator, 41(2), 224–230. DOI: 10.1177/0145721714567235 25614529 PMC4472382

[53] Markowitz, J. T., Garvey, K. C., & Laffel, L. M. (2015). Developmental changes in the roles of patients and families in type 1 diabetes management. Current Diabetes Reviews, 11(4), 231–238. DOI: 10.2174/1573399811666150421114146 25901503 PMC4826732

[54] Martinez, K., Frazer, S. F., Dempster, M., Hamill, A., Fleming, H., & McCorry, N. K. (2018). Psychological factors associated with diabetes self-management among adolescents with Type 1 diabetes: A systematic review. Journal of Health Psychology, 23(13), 1749–1765. DOI: 10.1177/1359105316669580 27663288

[55] McGrady, M. E., Laffel, L., Drotar, D., Repaske, D., & Hood, K. K. (2009). Depressive symptoms and glycemic control in adolescents with type 1 diabetes: Mediational role of blood glucose monitoring. Diabetes Care, 32, 804–806. DOI: 10.2337/dc08-2111 19228870 PMC2671131

[56] Monaghan, M., Clary, L., Mehta, P., Stern, A., Sharkey, C., Cogen, F. R., … Streisand, R. (2015). Checking in: A pilot of a physician-delivered intervention to increase parent-adolescent communication about blood glucose monitoring. Clinical Pediatrics, 54(14), 1346–1353. DOI: 10.1177/0009922815581833 25896723 PMC4615374

[57] Mulvaney, S. A., Anders, S., Smith, A. K., Pittel, E. J., & Johnson, K. B. (2012). A pilot test of a tailored mobile and web-based diabetes messaging system for adolescents. Journal of Telemedicine and Telecare, 18(2), 115–118. DOI: 10.1258/jtt.2011.111006 22383802 PMC4060437

[58] Murphy, H. R., Rayman, G., & Skinner, T. C. (2006). Psycho-educational interventions for children and young people with Type 1 diabetes. Diabetic Medicine, 23(9), 935–943. DOI: 10.1111/j.1464-5491.2006.01816.x 16922699

[59] Murphy, H. R., Wadham, C., Hassler-Hurst, J., Rayman, G., & Skinner, T. C. (2012). Randomized trial of a diabetes self-management education and family teamwork intervention in adolescents with type 1 diabetes. Diabetic Medicine, 29(8), e249–e254. DOI: 10.1111/j.1464-5491.2012.03683.x 22507080

[60] Murphy, H. R., Wadham, C., Rayman, G., & Skinner, T. C. (2007). Approaches to integrating paediatric diabetes care and structured education: Experiences from the Families, Adolescents, and Children’s Teamwork Study (FACTS). Diabetic Medicine, 24(11), 1261–1268. DOI: 10.1111/j.1464-5491.2007.02229.x 17894831

[61] Nansel, T. R., Iannotti, R. J., & Liu, A. (2012). Clinic-integrated behavioral intervention for families of youth with type 1 diabetes: Randomized clinical trial. Pediatrics, 129(4), e866–e873. DOI: 10.1542/peds.2011-2858 22392172 PMC3313642

[62] Nansel, T. R., Iannotti, R. J., Simons-Morton, B. G., Cox, C., Plotnick, L. P., Clark, L. M., & Zeitzoff, L. (2007). Diabetes personal trainer outcomes: Short-term and 1-year outcomes of a diabetes personal trainer intervention among youth with type 1 diabetes. Diabetes Care, 30(10), 2471–2477. DOI: 10.2337/dc06-2621 17620445 PMC2365717

[63] Nansel, T. R., Iannotti, R. J., Simons-Morton, B. G., Plotnick, L. P., Clark, L. M., & Zeitzoff, L. (2009). Long-term maintenance of treatment outcomes: Diabetes personal trainer intervention for youth with type 1 diabetes. Diabetes Care, 32(5), 807–809. DOI: 10.2337/dc08-1968 19208916 PMC2671090

[64] Nansel, T. R., Laffel, L. M. B., Haynie, D. L., Mehta, S. N., Lipsky, L. M., Volkening, L. K., Butler, D. A., Higgins, L. A., & Liu, A. (2015). Improving dietary quality in youth with type 1 diabetes: Randomized clinical trial of a family-based behavioral intervention. International Journal of Behavioral Nutrition and Physical Activity, 12(1), 58. DOI: 10.1186/s12966-015-0214-4 25952160 PMC4436744

[65] Nansel, T. R., Thomas, D. M., & Liu, A. (2015). Efficacy of a behavioral intervention for pediatric type 1 diabetes across income. American Journal of Preventive Medicine, 49(6), 930–934. DOI: 10.1016/j.amepre.2015.05.006 26231856 PMC4706073

[66] Naranjo, D., & Hood, K. (2013). Psychological challenges for children living with diabetes. Diabetes Voice, 58(S1), 38–40.

[67] Newton, K. H., Wiltshire, E. J., & Elley, C. R. (2009). Pedometers and text messaging to increase physical activity: Randomized controlled trial of adolescents with type 1 diabetes. Diabetes Care, 32(5), 813–815. DOI: 10.2337/dc08-1974 19228863 PMC2671105

[68] Newton, K. T., & Ashley, A. (2013). Pilot study of a web-based intervention for adolescents with type 1 diabetes. Journal of Telemedicine & Telecare, 19(8), 443–449. DOI: 10.1177/1357633X13512069 24197399

[69] Nicholas, D. B., Fellner, K. D., Frank, M., Small, M., Hetherington, R., Slater, R., & Daneman, D. (2012). Evaluation of an online education and support intervention for adolescents with diabetes. Social Work in Health Care, 51(9), 815–827. DOI: 10.1080/00981389.2012.699507 23078013

[70] Noyes, J., Allen, D., Carter, C., Edwards, D., Edwards, R. T., Russell, D., Russell, I. T., Spencer, L. H., Sylvestre, Y., Whitaker, R., Yeo, S. T., & Gregory, J. W. (2020). Standardised self-management kits for children with type 1 diabetes: Pragmatic randomised trial of effectiveness and cost-effectiveness. BMJ Open, 10(3), e032163. DOI: 10.1136/bmjopen-2019-032163 PMC7069268 32169923

[71] Pham, M. T., Rajić, A., Greig, J. D., Sargeant, J. M., Papadopoulos, A., & McEwen, S. A. (2014). A scoping review of scoping reviews: Advancing the approach and enhancing the consistency. Research Synthesis Methods, 5, 371–385. DOI: 10.1002/jrsm.1123 26052958 PMC4491356

[72] Price, K. J., Knowles, J. A., Wales, J. K. H., Heller, S., Eiser, C., & Freeman, J. V. (2016). Effectiveness of the Kids in Control of Food (KICk-OFF) structured education course for 11–16 year olds with type 1 diabetes. Diabetic Medicine, 33(2), 192–203. DOI: 10.1111/dme.12881 26248789

[73] Ramírez-Mendoza, F., González, J. E., Gasca, E., Camacho, M., Cruz, M. V., Caraveo, D., Velázquez, A., Cruz, Z., Segoviano, M., Romano, M., Diego, M., Made, A. M., León, D. C. de, Gay-Molina, J., & Prada, D. (2020). Time in range and HbA1C after 6 months with a multidisciplinary program for children and adolescents with diabetes mellitus, real world data from Mexico City. Pediatric Diabetes, 21(1), 61–68. DOI: 10.1111/pedi.12921 31584229 PMC6973224

[74] Serlachius, A. S., Scratch, S. E., Northam, E. A., Frydenberg, E., Lee, K. J., & Cameron, F. J. (2016). A randomized controlled trial of cognitive behaviour therapy to improve glycaemic control and psychosocial wellbeing in adolescents with type 1 diabetes. Journal of Health Psychology, 21(6), 1157–1169. DOI: 10.1177/1359105314547940 25213114

[75] Shalev, I., & Geffken, G. R. (2015). Use of self-regulation principles to improve adolescent treatment adherence to the medical regimen for diabetes. Journal of Psychotherapy Integration, 26(4), 366–377. DOI: 10.1037/int0000019

[76] Shaw, R. J. (2001). Treatment adherence in adolescents: Development and psychopathology. Clinical Child Psychology and Psychiatry, 6(1), 137–150. DOI: 10.1177/1359104501006001011

[77] Smart, A. (2006). A multi-dimensional model of clinical utility. International Journal of Quality in Health Care, 18(5), 377–382. DOI: 10.1093/intqhc/mzl034 16951425

[78] Spiegel, G., Bortsov, A., Bishop, F. K., Owen, D., Klingensmith, G. J., Mayer-Davis, E. J., & Maahs, D. M. (2012). Randomized nutrition education intervention to improve carbohydrate counting in adolescents with type 1 diabetes study: Is more intensive education needed? Journal of the Academy of Nutrition & Dietetics, 112(11), 1736–1746. DOI: 10.1016/j.jand.2012.06.001 22975086 PMC3487717

[79] Survonen, A., Salanterä, S., Näntö-Salonen, K., Sigurdardottir, A. K., & Suhonen, R. (2019). The psychosocial self-efficacy in adolescents with type 1 diabetes. Nursing Open, 6(2), 514–525. DOI: 10.1002/nop2.235 30918702 PMC6419123

[80] Taddeo, D., Egedy, M., & Frappier, J.-Y. (2008). Adherence to treatment in adolescents. Paediatric Child Health, 13(1), 19–24. DOI: 10.1093/pch/13.1.19 PMC2528818 19119348

[81] Tudur-Smith, C., Hopkins, C., Sydes, M. R., Woolfall, K., Clarke, M., Murray, G., & Williamson, P. (2015). How should individual participant data (IPD) from publicly funded clinical trials be shared? BMC Medicine, 13(298), 1–7. DOI: 10.1186/s12916-015-0532-z 26675031 PMC4682216

[82] Verbeek, S., Vos, R. C., Mul, D., & Houdijk, M. (2011). The influence of an education program on HbA1c-level of adolescents with type 1 diabetes mellitus: A retrospective study. Journal of Pediatric Endocrinology and Metabolism, 24(1–2), 15–19. DOI: 10.1515/jpem.2011.104 21528809

[83] Viklund, G., Ortqvist, E., & Wikblad, K. (2007). Assessment of an empowerment education programme. A randomized study in teenagers with diabetes. Diabetic Medicine, 24(5), 550–556. DOI: 10.1111/j.1464-5491.2007.02114.x 17367306

[84] Von Sengbusch, S., Muller-Godeffroy, E., Hager, S., Reintjes, R., Hiort, O., & Wagner, V. (2006). Mobile diabetes education and care: Intervention for children and young people with type 1 diabetes in rural areas of northern Germany. Diabetic Medicine, 23(2), 122–127. DOI: 10.1111/j.1464-5491.2005.01754.x 16433708

[85] Wade, D. T., & Halligan, P. W. (2017). The biopsychosocial model of illness: A model whose time has come. Clinical Rehabilitation, 31(8), 995–1004. DOI: 10.1177/0269215517709890 28730890

[86] Waller, H., Eiser, C., Knowles, J., Rogers, N., Wharmby, S., Heller, S., … Price, K. (2008). Pilot study of a novel educational programme for 11–16 year olds with type 1 diabetes mellitus: The KICk-OFF course. Archives of Disease in Childhood, 93(11), 927–931. DOI: 10.1136/adc.2007.132126 18676435

[87] Wang, Y. C., Stewart, S. M., Mackenzie, M., Nakonezny, P. A., Edwards, D., & White, P. C. (2010). A randomized controlled trial comparing motivational interviewing in education to structured diabetes education in teens with type 1 diabetes. Diabetes Care, 33(8), 1741–1743. DOI: 10.2337/dc10-0019 20484124 PMC2909053

[88] Weissberg-Benchell, J., Glasgow, A. M., Tynan, W. D., Wirtz, P., Turek, J., & Ward, J. (1995). Adolescent diabetes management and mismanagement. Diabetes Care, 18(1), 77–82. DOI: 10.2337/diacare.18.1.77 7698052

[89] Whittemore, R., Jaser, S. S., Jeon, S., Liberti, L., Delamater, A., Murphy, K., … Grey, M. (2012). An internet coping skills training program for youth with type 1 diabetes: Six-month outcomes. Nursing Research, 61(6), 395–404. DOI: 10.1097/NNR.0b013e3182690a29 22960587 PMC3623558

[90] Whittemore, R., Liberti, L. S., Jeon, S., Chao, A., Minges, K. E., Murphy, K., & Grey, M. (2016). Efficacy and implementation of an internet psychoeducational program for teens with type 1 diabetes. Pediatric Diabetes, 17(8), 567–575. DOI: 10.1111/pedi.12338 26611663 PMC4882266

[91] Wiebe, D. J., Berg, C. A., Mello, D., & Kelly, C. S. (2018). Self- and social-regulation in type 1 diabetes management during late adolescence and emerging adulthood. Current Diabetes Reports, 18(5), 23. DOI: 10.1007/s11892-018-0995-3 29564640

[92] World Health Organization. (2016). Global report on diabetes. Author. Retrieved from: http://apps.who.int/iris/bitstream/10665/204871/1/9789241565257_eng.pdf?ua=1

[93] Wysocki, T., Harris, M. A., Buckloh, L. M., Mertlich, D., Lochrie, A. S., Mauras, N., & White, N. H. (2007). Randomized trial of behavioral family systems therapy for diabetes: Maintenance of effects on diabetes outcomes in adolescents. Diabetes Care, 30(3), 555–560. DOI: 10.2337/dc06-1613 17327320

[94] Wysocki, T., Harris, M. A., Buckloh, L. M., Mertlich, D., Lochrie, A. S., Taylor, A., …White, N. H. (2006). Effects of behavioral family systems therapy for diabetes on adolescents’ family relationships, treatment adherence, and metabolic control. Journal of Pediatric Psychology, 31(9), 928–938. DOI: 10.1093/jpepsy/jsj098 16401678

[95] Wysocki, T., Harris, M. A., Buckloh, L. M., Mertlich, D., Lochrie, A. S., Taylor, A., …White, N. H. (2008). Randomized, controlled trial of behavioral family systems therapy for diabetes: Maintenance and generalization of effects on parent-adolescent communication. Behavior Therapy, 39(1), 33–46. DOI: 10.2337/dc06-1613 18328868

